# Society Proceedings

**Published:** 1898-12

**Authors:** 


### SOCIETY PROCEEDINGS.

### CHICAGO VETERINARY SOCIETY.

THE regular monthly meeting of the Society was held November 10th, President Robertson presiding. The following visitors and about twenty members were present: Drs. Boyd Baldwin, Walter E. Howe, and L. Enos Day. Dr. H. D. Paxson, U. of Pa., 1893, was admitted to membership. Owing to disagreeable weather, many refrained from attending.

The papers expected from Dr. Joseph Hughes, "Cases Met With in Practice," and reports of cases by Dr. John F. Ryan, were not presented, due in both instances to illness of the gentlemen. Matters, however, were mended by Dr. L. A. Merillat's response to President Robertson's call for a résumé of his paper on the "Action of Eserine," read before the U. S. V. M. A. The recitation brought out quite a lively discussion, following which Dr. Merillat called attention to Dr. Roscoe R. Bell's paper on "Acute Indigestion," commending it as worthy of careful perusal.

Dr. H. W. Hawley was next called upon and asked, "What is the Usual Length of Time for a Horse to go Sound Following the Operation of Neurectomy for Navicular Arthritis?" which brought out a very interesting discussion.

Dr. E. L. Quitman related a case of "Calcification of Parotid Gland and Steno's Duct," after which the discussion was closed and the following papers were announced for the December meeting. Dr. H. W. Hawley, "Lameness on Leading Out;" also, "Vices and their Relation to Soundness." Dr. A. M. Casper, "Temperature and its Relation to Soundness."

JOSEPH B. CLANCY,
Secretary.

*Report of cases and experiments discussed before the Chicago Veterinary Society, November* 10, 1898.

Dr. Merillat: During the past winter Dr. Reading and myself made several experiments on dogs at McKillip Veterinary College for the purpose of ascertaining the action of eserine. Eserine is an alkaloid and is described as a stimulant of the muscles of the bowels and, in fact, all muscular tissue. Through these experiments, however, we have shown that the action of eserine was purely upon the nerve-centres, and that instead of being a stimulant to the muscular coat of the bowels, it is no more than a depressant to the nerve-centres. Eserine depresses or paralyzes the nerves which stop the bowels, the inhibitory nerves, and by doing so rapidly empties them. In experimenting on horses that have paralyzed bowels we found that eserine was not active, but that it will act on the colon when there is marked peristalsis.

I may also mention the new treatment for acute indigestion recommended by Professor Bell, whose paper read at Omaha on "Acute Indigestion," is one of the best ever written on the subject. His treatment is very simple, and consists in the administration of large amounts of dry animal charcoal. It is well known to the chemist that charcoal will absorb about four hundred times its volume of gas; if, therefore, a great amount, say eight or ten ounces, be administered dry, it will rapidly absorb any gas in the stomach. Dr. Bell reports great success in cases of acute indigestion by such treatment. He emphasizes the fact that if you wish to get any benefit from this treatment the charcoal must be administered dry and in combination with sodium bicarbonate. You may combine other treatment, as the administration of the charcoal does not in any way interfere with other agents used for this condition. I think it is certainly a treatment that every city practitioner should try.

Dr. Campbell: It seems to me that as soon as the capsule in which the charcoal is administered gets wet the charcoal gets wet also, and it would not have the desired effect.

Dr. Merillat: As charcoal absorbs about four hundred times its volume of gas, as stated before, one ounce would be enough to have the desired effect if it did not moisten in the stomach; but, instead, he administers a much larger amount, so that while a portion of it gets moist, the balance remains dry and absorbs the gas.

Dr. Quitman: Regarding the action of eserine, I am very much pleased to hear of the experiments that Dr. Merillat has made, inasmuch as they bear out my idea from a theoretical standpoint. We know that some drugs have the power of paralyzing the motor nerves. I worked it out in the same way. In regard to its indications and use, I would dislike very much to be without it, as I am usually successful with it. We have to select our cases to administer it. Where there is paralysis of the bowels, sometimes you get good action, even then, by giving it in small doses, repeated. I get eserine in one-and-a-half grain sealed tubes. It becomes moist on instant exposure; therefore I buy it in one-dose tubes, so as to keep it fresh. The tablets, I think, are worthless, because such a drug cannot be entirely protected from atmospheric moisture.

Dr. Hawley: This discussion calls to my mind a case, and it does not bear out the theory of either Drs. Merillat or Quitman. In 1891, while I was assistant to Dr. Hughes, a horse weighing about 1200 pounds was brought to the college. It was apparently a case of ordinary impaction. I do not remember all of the treatment, but I do remember that he received, first, a pint of linseed-oil, which was followed with a half-pint every four hours for eleven days.

Dr. Hughes was at that time much opposed to administering eserine, but finally consented. A grain and a half of eserine was injected, and we had a violent action of the bowels in twenty minutes, presumably one-half bushel of feces, and the horse finally recovered.

Dr. Merillat: I think this is a nice case that demonstrates the theory of Dr. Quitman and my own. Dr. Hughes probably had the horse in good condition to get the action of eserine. The treatment that Dr. Hughes had previously given was just commencing to take action on the bowels and the administration of eserine had the desired effect.

Dr. Campbell: I had a case similar to that of Dr. Hawley's. After administering eserine, although it had very violent action, the horse died two days afterward.

Dr. Quitman: That recalls to my mind a case I had some time ago. It apparently seemed to be an ordinary case of colic. A colic-drench and purgative ball were administered without any good effect. The sounds in the bowels were normal. I repeated the purgative, gave injections of eserine, and there was absolutely no result until the fourteenth day, when the horse passed a few pellets. I did not know what else to do, and came to the conclusion that there was an intestinal obstruction, and advised the owner to turn the horse out. On the thirty-first day enteritis set in, and the horse died. I went out to the country and held a postmortem, and I found a calculus that weighed about one pound and two ounces, rounded in shape, and the pouch in which it was developing very much thickened. It was located about two feet back of the commencement of the single colon. Another peculiar feature I noticed was that the digesting portion of the stomach was atrophied and looked like parchment. Of course, cases where there is obstruction of the bowels cannot be helped by eserine.

I also have a case of lameness that I would like to have some one help me out on. She is a driving mare, and starts out apparently well, and after she is driven five or six miles and is turned around starting for home she starts to go lame, then suddenly goes sound again. It is in the off front leg. She had been going that way for five or six days, when the owner asked me to examine her. I examined the leg in question, taking the owner's word for it—as he is a very good horseman—as to which leg she was lame in. I rode behind her, and drove about eight miles, then let her stand quite awhile, but could not discern any lameness. I prescribed an ointment of cocaine and morphine dissolved in oleic acid and mixed with oleate of mercury and lanolin, but this did not overcome the lameness. I have not seen the mare since, but I know the owner speaks the truth because he is not a man to fool me. Now, I do not know what to make of it. I am handicapped somewhat in not seeing the mare. All I noticed when I drove her and examined her foot was what seemed to be a small splint.

Dr. Merillat: How long had these symptoms been manifest?

Dr. Quitman: About four weeks.

The Chairman: Will Dr. Hawley favor us with a few remarks?

Dr. Hawley: I would like to ask a few questions in regard to neurectomy. How long does an animal usually go sound following successful operation for navicular arthritis?

Dr. Quitman: I know of a case that went sound for seven years.

Dr. Allen: I know of one that I operated upon—high operation—ten years ago, and he is sound to-day.

Dr. Merillat: The length of relief from the operation depends, in my opinion, upon the work that they have to do afterward. The average in lower plantar operation is two years. High neurectomy is an operation that I would recommend only as a last resort for navicular disease, as the danger is too great, though in many cases it may prove successful. Meso-neurectomy is very efficient in tendonitis. In all such cases it gives prompt relief, and often completely straightens the tendon.

Another operation that is effectual in spavin is post-tibial neurectomy. It is a sure relief to ringbone where there is no mechanical obstruction. All these neurectomies require a little courage, but I think we would advance our profession a great deal if they were universally adopted.

Dr. Allen: I am much in favor of the high operation. For about ten years that I have been in active practice I have performed three or four of the lower and about seventy of the higher operations, and I have yet to lose my first case. As to post-tibial neurectomy for ringbone, I always perform high operation with good results, and do not see the necessity of going as high as Dr. M. recommends.

Dr. Hawley: In absolute navicular arthritis I do not see the necessity of performing the high operation.

### MONTREAL VETERINARY MEDICAL ASSOCIATION.

THE regular meeting was held in the Library of the College on the evening of November 3d. The President, Dr. Adami, occupied the chair; there was a fair attendance of members, supplemented by the presence of the Hon. President, Dr. D. McEachran, Prof. Mills, Dr. Alloway, Dr. Gunn, Dr. Moore, and Dr. Sugden.

After disposing of routine business, the chairman called upon Mr. Kato, who then presented to the Society his essay on "Eclampsia." The word eclampsia (derived from the Greek), meaning to shine or burst forth, was used by some authors at a very remote period, and is now the term commonly applied to cramp or convulsion of involuntary muscles, ocurring after parturition. It is generally believed that the disease attacks mostly bitches. Opinion as to its cause is very varied, but that the symptoms result from a disordered nervous system is beyond question; but it is very difficult to determine the cause of this condition. Like the other tissues of the body, the nerves undergo a process of degeneration and repair, and it is possible that in this disease the materials proper for its repair are not circulating in the bloodvessels, and thus bring about this disordered action of the nervous system, which system must always be considered as an important factor, especially in such diseases as eclampsia. The dogs most susceptible were Skye-terriers, Yorkshire spaniels, and collies, and the various toys. This increased susceptibility is probably due to their excessive sensibility to external influences, such as excitement, worry, etc. Mr. Kato then described a case which came under his own notice. In spite of the severe nature of the disease, consciousness is not lost, and any one may often observe an animal try to wag its tail when called by name. An attack may last for twenty-four hours or more, but with varying intensity during that period. In mild cases recovery takes place without treatment. On the other hand, if the attack be an acute one the patient may fall into a comatose condition and die in a very short time. A sedative treatment is indicated, and for this purpose we may use hypodermics of morphine, the administration of chloral hydrate, bromide of potassium, or inhalations of chloroform. Warm baths, followed by massage, often relieve spasms and release these muscles. An oleaginous laxative may always be given with benefit. Mr. Kato, in conclusion, said that a dark, quiet, well-ventilated place was infinitely better for the patient than noisy surroundings, and that the prognosis was fairly favorable, provided that the patient be attended at the proper time with energetic measures.

Before making any remarks on the subject of eclampsia, Dr. McEachran complimented the essayist on the excellent paper which he had read, and upon the exceedingly good manner in which it was delivered, in what was to Mr. Kato a foreign language. Dr. McEachran has most frequently seen eclampsia in bitches left with too many pups to suckle, the excessive secretion of milk causing a depletion of the blood, which resulted in an anæmic condition of the various nerve-centres. Treatment was, as a rule, attended with success, and the disease need not be looked upon as a formidable one by the young practitioner.

Before closing his remarks Dr. McEachran extended, on behalf of the Society, a very hearty welcome to Dr. Alloway, who subsequently made a few remarks, commencing by recalling his early days in college, he having purchased some thirty-one years ago the animal whose skeleton now adorns our lecture-room. Dr. Alloway, who has recently returned from the Western States, spoke of the comparatively high value of dogs in those regions, and pointed out to the students the necessity of their giving up the old habit of devoting all their attention to the study of the equine race.

Dr. Mills also complimented Mr. Kato, and, in continuing, said that he had found the Japanese spaniels more delicate than any other of the toy breed. He had never had a case of eclampsia in his own kennels, which fortunate state of affairs he attributed to his bitches being regularly exercised and never being allowed to suckle too many pups.

Before considering the subject of eclampsia, Dr. Adami expressed his pleasure at being with the Society once more. Eclampsia of the bitch differed very widely from eclampsia of the human subject, although in both the condition was closely allied with the puerperal state; in the human being it was a far more serious disease. Observations now seemed to point to some alteration of the blood as the path along which we must search for a solution of the problem. Patches of coagulated necrosis were commonly seen in the liver, more frequently in this than any other disease. These might possibly be due to thrombosis; so far no bacteria had been found in them, and it is supposed that this condition may be produced by toxins. Hitherto all attempts at finding a specific microbe had failed.

Mr. Groves then reported the following interesting case: The subject, a male dog, ten years old, of spaniel breed, brought to the college for treatment. The dog was unable to walk in a straight line, often falling down, and lying down more than usual. This condition was noticed for about two weeks. The symptoms presented were a general debilitated condition, coat rough and dry; the tongue protruding from the left side of the mouth, the neck straight and sometimes inclined to the left; the body resembled the segment of a large circle, with its centre on the right side; lameness in the right foreleg, and general paresis. Diagnosis: pressure on base of the brain, probably a tumor; prognosis, unfavorable. Treatment useless; but, as the owner desired that we should do something, he was given nux vomica, iodide of potash, and gentian. Two months afterward the animal was brought back to be destroyed. The symptoms were much aggravated, the dog lying most of the time, but he could get up, and when he tried to go fast fell down; when standing, would brace himself against some object, and when walking would walk almost sideways, always going to the right; his head was turned on its axis from the right to left, the right side being the highest; this was not continually the case, for he would at times hold his head straight; his vocal power was lost, and he had difficulty in swallowing; sight was not impaired. The dog was chloroformed and postmortem held, which resulted as follows: The various organs of the body were found to be normal, but on the inferior part of the medulla there was found a tumor about the size of a marble. It was attached closely to the wall of the foramen magnum, so that it had to be separated with a knife. It was semicircular in form, with a broad base.

Dr. Gunn considered this an operable case, and a surgeon would have been guided to the right lobe of the cerebellum as the seat of tumor. The diagnosis would have been wrong, but the surgeon's incisions would have been over the tumor, which could have been shelled out with a possible good result. The recognized symptoms of the disease of the cerebellum are almost exactly those given by Mr. Groves, viz.: (*a*) rotation of the body away from the line on the opposite side to tumor; (*b*) the neck was twisted and chin turned to side of tumor; (*c*) bending of the body, the concavity being on the affected side; (*d*) monobrachial paralysis, which is not very definite in this case; (*e*) eye-symptoms were not much observed in this dog. The relative size of the pupils was different, but there seems to have been no nystagmus nor strabismus, as is common in cerebellar trouble. Convulsive and choreic seizures and vomiting are reported as absent here, but usual in pure cerebellar trouble.

Dr. Mills regretted that the symptoms produced in the eye and ear were not more closely noted, and said that the views held upon functions of the cerebellum were very divergent. He had, unfortunately, never seen a case of cerebellar disease in the dog.

After a few words from the chairman, the meeting adjourned.

James McGregor,
Secretary-Treasurer.

### VERMONT STATE VETERINARY MEDICAL ASSOCIATION.

The first meeting was held at Montpelier, October 25, 1898. The meeting was well attended, and was a success as a primary organization. No business was transacted, with the exception ot adoption of by-laws and election of officers, as follows: President, F. A. Rich, Burlington; Vice-President, H. W. Burgess, Bennington; Secretary, Ion W. Parks; Treasurer, H. Buss, St. Johnsbury; Executive Committee, G. A. Miller, Burlington; J. F. Page, Manchester Centre; E. W. Culley, Morrisville; H. Buss, St. Johnsbury; J. C. Parker, St. Albans; W. L. Adams, Hardwick. Committee on Credentials: W. H. Burgess, Bennington; C. L. Morrin, St. Albans; A. B. Gay, Randolph.

### KEYSTONE VETERINARY MEDICAL ASSOCIATION APPOINTMENTS, 1898–1899.

Committee on Programme: W. L. Rhoads, chairman; J. J. Repp and J. W. Adams.

Committee on History: Charles T. Goentner, chairman; W. Horace Hoskins and W. B. E. Miller.

Committee on Legislation: W. Horace Hoskins, chairman; F. S. Allen, W. S. Kooker, James B. Rayner, and W. H. Ridge.

Committee on Army Legislation: C. J. Marshall, chairman; Francis Bridge, H. P. Eves.

### TENNESSEE VETERINARY MEDICAL ASSOCIATION.

The third annual meeting was held in Chattanooga on Monday, October 24, 1898. The President, Dr. Joseph M. Good, occupied the chair, and in his opening address touched briefly on many subjects of importance to the veterinarians throughout the State. He referred to the benefits which had been derived from such an organization, and outlined the good results which we might hope for in the future, and showed that the veterinary profession in the State was on a better standing now than at any time in the past. The attendance, considering the limited number of qualified men in the State, was very gratifying, as the following members answered to their names: Drs. Joseph M. Good, H. D. Fenimore, J. W. Scheibler, T. W. Scott, W. C. Rayen, G. B. Blackman, G. R. White, Joseph Plaskett. The Secretary read the minutes of the last meeting, and after some slight alterations these were adopted.

The Committee on Legislation presented a report stating that during the coming meeting of the State Legislature a strong effort will be put forth to obtain the passage of a bill regulating the practice of veterinary medicine and surgery within the bounds of the State. This bill will compel all qualified practitioners to register, and will give all unqualified men who have been practising for five consecutive years in the State the privilege of registering and continuing their practice. It is also intended to create a State Veterinary Examining Board, which shall be appointed by the Governor from members of the Tennessee Veterinary Medical Association. It seems to be the opinion of the members that if a proper effort were made the passage of such a bill could be obtained; and each one pledged himself to use his best efforts in that direction.

The Committee on Resolutions presented a report deploring the fact that the cities of Memphis, Chattanooga, and Knoxville had no system of meat- and milk-inspection under the supervision of a man specially qualified for such duties, thereby endangering the health and lives of their citizens, and it was resolved that such a condition of affairs necessitated a system of inspection as conducted by other cities in the country, which system is directly under the control of qualified veterinarians specially educated for such positions. It was also resolved that the Tennessee Veterinary Medical Association do most earnestly coöperate with the Legislative Committee of the American Veterinary Medical Association in its efforts to obtain recognition of and commission for the army veterinarian.

The reports of the Committees on Legislation and Resolutions were adopted. The next business being the reading of papers, the President called on Dr. Blackman, who responded with a thoughtful and well-prepared paper on the subject, "Veterinarians as Sanitarians." All the members joined in the discussion which followed, and it proved to be an interesting and instructive topic.

He was followed by Dr. White, whose subject was "Municipal Meat-inspection." Dr. White's position as meat-inspector for the city of Nashville enabled him to handle the subject in a competent and practical manner, and he plainly illustrated the necessity for a system of inspection in all the larger towns of the State.

Dr. Plaskett came next with a paper on "Trismus in Horses," a disease which does not appear to be described in the text-books or in veterinary literature. It is quite prevalent throughout the South, and in the discussion which followed every member had something to say on the subject in some of its aspects.

Dr. Fenimore followed with an informal talk on the subject of "Vilde und Rinderseuche." He described an outbreak which had occurred on the

State farm at Knoxville and the difficulty which had been experienced in properly diagnosing it. He described the symptoms, attempts at treatment, etc., and for further information referred the members to a paper which he had prepared and which was read at the annual meeting of the National Association. The next business being the election of officers, the following were elected for the ensuing year: President, Dr. H. D. Fenimore; First Vice-President, Dr. T. W. Scott; Second Vice-President, Dr. R. E. Collins. Dr. Plaskett was reëlected as Secretary, and Dr. Blackman was elected Treasurer. The time and place of the next meeting was left to the Executive Committee, composed of the officers of the Association, and, there being no other business, the meeting adjourned.

JOSEPH PLASKETT, D.V.S.,
Secretary.

### SEVENTH INTERNATIONAL CONGRESS OF VETERINARY SURGEONS, BADEN-BADEN, AUGUST 9 to 14, 1899.

THE following gentlemen have undertaken to draw up reports of the subjects already announced for transaction and discussion by the congress.

a. *Precautionary measures against the spread of epidemic diseases in consequence of international trade in animals.*

Reporters: Cope, President of the veterinary surgery section of the Chamber of Agriculture, London; Dr. Hutyra, Professor and Head of the Veterinary Academy in Budapest; Leblanc, veterinary surgeon for epidemic diseases, Member of the Academy of Medicine in Paris; Vollers, Government veterinary surgeon in Hamburg. (Swiss reporters are still wanting.)

b (1). *The prevention of tuberculosis among domestic animals.*

Reporters: Dr. Bang, Professor at the Veterinary College in Copenhagen; Dr. Siedamgrotzky, Privy Medical Councillor, Professor in the Royal Veterinary College in Dresden, district veterinary surgeon in the Kingdom of Saxony; Dr. Stubbe, Veterinary Inspector of the Ministry of Agriculture in Brussels.

(2). *The prevention of the use of the flesh and milk of animals suffering from tuberculosis.*

Reporters: Butel, veterinary surgeon to the slaughter-house in Meaux; de Yong, Government veterinary surgeon in Leyden; Dr. Ostertag, Professor at the Royal Veterinary College in Berlin.

(3). *The latest suggestions for an effectual meat-inspection.*

Reporters: Dr. Edelmann, superintendent of meat-inspection in Dresden; Kjerulf, Government veterinary surgeon in Stockholm; Postolka, Imperial official veterinary surgeon in Vienna.

c. *The prevention of foot-and-mouth-disease.*

Reporters: Paul Cagny, veterinary surgeon in Senlis; Cope, as above, of London; Dr. Dammann, Privy and Medical Councillor, Professor and Head of the Royal Veterinary College in Hanover; Dr. Furtuna, President of the Veterinary Office in Bucharest; Hafner, Councillor and veterinary referee of the Grand Ducal Ministry of the Interior in Carlsruhe; Hess, Professor at the Veterinary School in Berne; Lindguist, Professor and Head of the Veterinary College in Stockholm; Dr. Wirtz, Professor and Head of the Veterinary College in Utrecht (has not yet given a decided answer in the affirmative).

*d. The prevention of swine fever.*

Reporters: Leclainche, Professor in the Veterinary School at Toulouse; Dr. Lorenz, Grand Ducal medical officer in Darmstadt; Dr. Perroncito, Professor at the Veterinary Academy in Turin.

*e. The extension of veterinary instruction, especially by the establishment of institutions for experiments in diseases and by founding chairs of comparative medicine in colleges for veterinary surgery.*

Reporters: Degive, Professor and Head of the Veterinary College in Brussels; Dr. Kitt, Professor at the Royal Veterinary College in Munich; Dr. Malkmus, Professor at the Royal Veterinary College in Hanover; Dr. Nocard, Professor at the Veterinary College of Alfort, Paris, Member of the Academy of Medicine; Dr. Raupach, Councillor, Professor and Head of the Imperial Veterinary Institute in Dorpat; Dr. Schütz, Privy Councillor, Professor at the Royal Veterinary College in Berlin.

*f. Conclusion of the work of drawing up a universal anatomical nomenclature in veterinary medicine in accordance with resolutions passed by the Sixth Congress.*

Reporters: Dr. Ellenberger, Medical Councillor, Professor at the Royal Veterinary College in Dresden; Dr. Sussdorf, Professor at the Royal Veterinary College in Stuttgart.

*g. Veterinary officials.*

Reporter: Dr. Lydtin, Privy Councillor in Baden-Baden.

The majority of reporters have agreed to send in their reports by January, 1899. The translation and printing of the reports will take about two or three months, though single reports may be ready during the first quarter of 1899.

In order that the gentlemen who wish to take part in the work of the Congress, or who take an interest in this work, may receive the reports and other publications of the Congress at the earliest possible date, it is requested that applications for membership shall be sent in at once or, at the latest, by March 31st next year. This is to be done by sending the member's fee of $3.00 to the Filiale der Rheinischen Credit-Bank in Baden-Baden. Gentlemen who have become members, even if unable personally to be present at the Congress, will receive by post, free of charge, all publications, including the General Report of the proceedings. Those gentlemen who do not enter their names until the opening of the Congress will only receive the publications supplementarily.

The Committee of Management begs to draw attention to the fact that applications for apartments and rooms in boarding-houses can already be received by the Lodgings Committee, Lichtenthalerstrasse 9 I Stock, Baden-Baden.

Professor Noyer, of Berne, General Secretary of the Sixth International Congress of Veterinary Surgeons, Mr. Siegen, of Luxemburg, Government veterinary surgeon, Mr. Haas, of Metz, district veterinary surgeon, President of the Veterinary Society of Alsace-Lorraine and Mr. Zundel, of Mulhausen, Alsace, district veterinary surgeon, have kindly undertaken to act as interpreters from German into French and *vice versa.* The interpreter for English will not be decided upon until an adequate number of English-speaking members is announced.

The Committee of Management: Dr. Casper Hoechst, Secretary; Dr. Lydtin, Baden-Baden, President.

BADEN-BADEN, November 3, 1898.

### ONTARIO VETERINARY COLLEGE, VETERINARY MEDICAL SOCIETY.

THE first meeting of the Veterinary Medical Society of this College was held on the evening of October 14th, Prof. A. Smith, F.R.C.V.S., the Principal, presiding. Mr. Wesley M. Goff read an excellent essay on "Bacteria." Mr. G. Jerome brought forward a carefully-prepared paper on the "Progress of Veterinary Science." Mr. F. J. Kerwan read a paper on the "Examination of Horses for Soundness," and Mr. J. Lea Shorey read a communication on "Neurectomy." The discussions following each paper were animated and interesting.

The second meeting was held on the evening of October 21st. Mr. Wentzell read an exhaustive paper on "Open-joint." Mr. W. A. Sproule read a paper on "Castration," describing different methods of operating; and Mr. Charles Manning read a good paper on "Parturient Apoplexy." The papers were ably discussed by several of the members of the senior class, the one on parturient apoplexy eliciting an interesting discussion on the new treatment of that disease, called "Schmidt's treatment," and its cause ascribed to toxins developed in the mammary gland at that time.

These meetings are held weekly, and the discussions arising at them must prove of much benefit to the students attending the college.

W. M. GOFF,
Secretary.

### VETERINARY MEDICAL SOCIETY, UNIVERSITY OF PENNSYLVANIA.

THE fourth regular meeting of the year was called to order November 18th, at 8 o'clock. Mr. Taylor was appointed critic. After the transaction of the business pertaining to the welfare of the Society and its members, the literary programme of the evening was next in order, and the Society had the pleasure of being addressed by its honorary president, Dr. John W. Adams, his subject being "Flat Worms of Domestic Animals." The enthusiasm shown upon his being introduced, and likewise his lecture, clearly demonstrated the fact that the members were greatly pleased with it. An important feature of his discourse were the charts he used in making his subject better understood. A vote of thanks was extended to Dr. Adams.

Meeting adjourned at 9.30 P.M.

The fifth regular meeting of the year was called to order December 2d, at 8 o'clock. Mr. Cheney was appointed critic. This meeting was devoted to the consideration of the annual banquet of the Association and the reports of the various committees having in charge the several features of the annual occasion. The names of Messrs. Horner, Bassler, Hart, and Sidel were proposed and duly elected to membership.

The meeting adjourned at 9.50 P.M.

L. A. NOLAN,
Secretary.

